# Impact of pay-for-performance on hospital readmissions in Lebanon: an ARIMA-based intervention analysis using routine data

**DOI:** 10.1186/s12913-024-12045-1

**Published:** 2024-12-05

**Authors:** Jade Khalife, Walid Ammar, Fadi El-Jardali, Maria Emmelin, Björn Ekman

**Affiliations:** 1 Department of Clinical Sciences, Malmö, Faculty of Medicine, Lund University, Malmö, Sweden; 2 Higher Institute of Public Health, Faculty of Medicine, Saint Joseph University of Beirut, Beirut, Lebanon; 3 Department of Health Research Methods, Evidence, and Impact, McMaster University, Hamilton, ON Canada; 4 Department of Health Management and Policy, Faculty of Health Sciences, American University of Beirut, Beirut, Lebanon

**Keywords:** Readmission, Hospital, Pay-for-performance, Impact, Lebanon, Outcomes, Quality, ARIMA, LMIC

## Abstract

**Background:**

The objective of this paper was to estimate the impact of country-wide hospital pay-for-performance on readmissions for a set of common conditions in Lebanon.

**Methods:**

This retrospective cohort study included all hospitalizations under the coverage of the Ministry of Public Health in Lebanon between 2011 and 2019. We calculated 30-day all-cause readmissions following general, pneumonia, cholecystectomy and stroke cases. We used an interrupted time series design, including the use of AutoRegressive Integrated Moving Average models. This nationwide study including 1,333,691 hospitalizations was undertaken in Lebanon, using hospitalizations at about 140 private and public hospitals contracted by the Ministry. The participants included citizens across all ages under the Ministry’s coverage (52% of citizens). The intervention was the engagement of hospital leaders by the Ministry, informing them of the addition of a readmissions component to the ongoing pay-for-performance initiative. Engagement participants included hospital directors and managers, and the leadership of the Syndicate of Private Hospitals. The main outcome measure was age-adjusted monthly all-cause readmission rates for each of general, pneumonia, cholecystectomy and stroke cases. We also assessed for change in readmissions for three conditions not included in the intervention (myocardial infarction, cataract surgery and appendectomy).

**Results:**

Across 2011–2019, the overall readmission rates were 6.00% (SD 0.24%) for general readmissions, 5.06% (SD 0.22%) for pneumonia, 2.54% (SD 0.16%) for cholecystectomy, and 6.55% (SD 0.25%) for stroke. Using ARIMA models we found a relative percentage decrease in mean monthly readmissions in the post-intervention period for cholecystectomy (5.9%; CI 0.1%-11.8%) and stroke (13.6%; CI 3.1%-24.2%). There was no evidence of intervention impact on pneumonia and general readmissions, both overall and among small, medium and large hospitals. There was also no evidence of change in non-P4P readmissions of myocardial infarction, cataract surgery and appendectomy.

**Conclusions:**

Including readmissions within pay-for-performance has the potential to improve hospital performance and patient outcomes, even in countries with more limited resources. Effects may vary across conditions, indicating the need for careful design and understanding of the particular context, both with respect to implementation and to evaluation of impact.

**Supplementary Information:**

The online version contains supplementary material available at 10.1186/s12913-024-12045-1.

## Introduction

The Lebanese Ministry of Public Health (MoPH) established a pay-for-performance (P4P) initiative in 2014, to incentivize hospitals to improve their performance across select components [1]. The main components were on patient satisfaction, hospital casemix and accreditation. This determined the total hospital performance score (TPS), which was used to categorize about 140 hospitals across three reimbursement tiers. An evaluation of the 2014 intervention found that it improved healthcare effectiveness by reducing unnecessary hospitalizations, and led to better medical discharge coding practices [1]. To incentivize hospitals to reduce readmissions, a new component on hospital readmission rates was announced by the MoPH in 2018, and implemented in the following year. This included readmissions for four conditions: general, pneumonia, stroke and cholecystectomy cases.

Hospital readmission has been commonly used in assessing the performance of hospitals and health systems, usually within a quality of care framework [2]. They also have a direct impact on patients themselves. Hospital readmissions are frequent, costly and sometimes life-threatening [3]. Several factors may influence the risk of readmission, some of which are not modifiable. These include factors at the patient, hospital and community levels. The most notable patient characteristics include age, medical condition or procedure, and comorbidities [4–8]. Hospital treatment quality and discharge instructions (and compliance) have a major role in readmissions, while the role of hospital size, volume and geography are less understood [8–12]. There is also a major role of community-level factors, such as socioeconomic status and poverty [4, 6].

### Linking readmissions to financial incentives

Reducing readmissions has been considered an important health policy goal, with some countries setting national-level policies, including Denmark, England, Germany and the United States [13]. In England and the US, these have been linked to financial incentives, within a P4P framework.

The most prominent national-level experience has been the Hospital Readmission Reduction Program (HRRP) in the United States, enacted by the Patient Protection and Affordable Care Act of 2010 [14]. HRRP’s first iteration targeted readmissions following acute myocardial infarction, heart failure and pneumonia. Penalties were applied from 2012 onwards on hospitals with higher than average readmission rates, initially at 1% of hospital reimbursement costs, and subsequently reaching up to 3%. The program later expanded to include Chronic Obstructive Pulmonary Disease (COPD), coronary artery bypass graft surgery, and hip and knee replacement surgery. Evaluations of the first HRRP iteration were generally favorable, with decreased readmission rates across all three conditions, hundreds of millions of dollars in healthcare savings, and no increased mortality [15–17]. However, some of the early decreases were not sustained. Some studies suggested HRRP may have led to unintended consequences such as increased post-discharge mortality for heart failure and pneumonia [14, 18]. This has led to considerable debate among researchers and policymakers, as well as important lessons learned [19].

In contrast to the HRRP, the Hip Fracture Best Practice Tariff (BPT) in England had a simpler P4P design, focusing on a few high-value outcomes. Implementation of the BPT was followed with decreased mortality among elderly patients with hip fracture, as well as the reversal of a previously increasing trend of readmissions [20].

### Limited resource countries’ challenges in tackling readmissions

Despite increased literature in the past decade on incentivizing readmission reduction in the US, similar national-level initiatives are uncommon. Contextual differences may expectedly be greater for countries with more limited resources to systematically collect data for tracking readmissions. This may include absence of computerized records at hospital level, weak medical coding capacity and absence of a harmonized database at regional or national levels.

### Purpose of this paper

The purpose of this article is to estimate the impact of the 2018 iteration of the MoPH P4P initiative on readmissions of general, pneumonia, stroke and cholecystectomy cases. In particular, we compare readmission rates before and after the announcement of the P4P component on readmissions, and compare to readmission rates of conditions not included in the P4P intervention (myocardial infarction, cataract surgery and appendectomy). We also analyze heterogeneous changes across hospital sizes, and explain the rationale and algorithms used to determine readmissions.

### Intervention

The MoPH engaged hospital leaders to inform them on the addition of a readmissions component to the ongoing pay-for-performance initiative. This was undertaken through two stakeholder engagement events, in January and March 2018, which centered on the policy and the technical levels respectively. Participants included hospital directors, quality managers, financial managers and the leadership of the Syndicate of Private Hospitals. Discussions involved the selection, calculation and weighing of performance components, including a new component on readmissions. Participants were informed that the readmission component would be comprised of general, pneumonia, stroke and cholecystectomy readmissions. A distinction is made between the impact of the announcement (evaluated in this paper) which represents an early stage in its roll out, and the eventual full implementation of P4P. It is common for hospitals to begin adapting their policies and procedures once new incentives are announced, even before formal implementation [21, 22]. The announcement acts as an informational intervention, conveying the new incentives, while the impact of full implementation may involve other factors, such as changes in financial flows.

## Methods

### Overall study design

We use an interrupted time series (ITS) design with an intervention analysis. This was necessarily determined by the availability of multi-year data from the MoPH hospitalization database coupled with the inability to randomize the intervention (MoPH legal regulations). In such circumstances, a valid inference of the causal relationship between intervention and outcome requires a well-designed time series experiment ([23], p. 2). We use a historical control, by categorizing pre- and post-intervention data-points within the same population. We chose an ITS design because it captures detailed temporal trends, including seasonality and month-to-month variation, which would be lost with the broad pre- and post-intervention averaging of Difference-in-Differences (DID). Additionally, DID requires a control group, which is difficult to establish in this case, while ITS effectively uses the hospitals' own pre-intervention data as a self-control.

### Study setting

Lebanon is an Eastern Mediterranean country with a population of about 6.8 million people, including two million refugees. The MoPH contracts about 140 private and public hospitals to provide coverage for 52% of citizens, who otherwise lack formal health insurance [1]. Hospitals are reimbursed for medical hospitalizations using a fee-for-service structure, and for surgical procedures using a predefined flat rate. This has been further described elsewhere [1, 24]. The reimbursement structure is the same across medical hospitalizations included in the study (fee-for-service for stroke, pneumonia, myocardial infarction) and across surgical procedures (flat-fee for cholecystectomy, cataract surgery, appendectomy), while general cases included all surgical and medical hospitalizations.

### Data collection and preparation

Administrative data can accurately capture all-cause readmissions [25]. Data was extracted from the MoPH hospitalization database by the Information Technologies Department. This included all cases under MoPH coverage between January 2011 and December 2019. The extracted data was provided in an annually segregated manner by case type (medical, surgical or intensive-care). Each file contained anonymized patient identifiers, with fields including admission and discharge dates, hospitalization record number, case identifier, age, sex, hospital code, medical code (ICD10) and/or surgical procedure code (CPT), and procedure order. The total dataset was comprised of 1,333,691 hospitalizations.

### Algorithms for readmission calculation

We developed case definitions for each of the readmission indicators, based on a review of the literature. Using these, algorithms were coded for each of the calculation of age-adjusted readmission rates across the four conditions, using Stata v.16 software. Monthly readmission rates at national level were calculated for 11 months per year, excluding December (see Additional file 1, Supplement A). This involved aggregating monthly data on readmission rates for each hospital under the MoPH. A total of 99 monthly data points were generated, including 80 pre-intervention data points and 19 post-intervention data points (after March 2018). To improve interpretability, linear interpolation was used to populate December values between 2011 and 2018.

For all conditions, a readmission was identified as a patient having been readmitted within 30 days from previous hospitalization discharge, regardless of readmission cause (i.e. any-cause), unless otherwise specified (see Additional file 1, Supplement A). A readmission was attributed to the index hospital (the hospital where the initial admission occurred), regardless of whether the patient was readmitted to the same hospital or a different one. General cases included all medical and surgical hospitalizations, unless otherwise specified. Patients with multiple readmissions per year had only the first readmission counted as such, to limit the effect of outliers due to patients with high comorbidities.

The MoPH included general readmissions in the P4P initiative to encourage hospitals to address broader care pathways. This approach was designed to prevent the unintended consequence of improving targeted areas at the expense of non-targeted or broader readmissions. Since general readmissions were a component of the MoPH P4P, we have included them in our analysis.

### Age adjustment of readmission rates

We used direct adjustment for age on the crude readmission rates. The 2015 denominator population was used as the standard reference for readmission data across 2011–2019. Each month of 2015 was used as the standard population for the corresponding month of other years. This was undertaken since monthly readmission rates were desired, and we expect the proportion of ages to vary across different months according to seasonal disease patterns. We used six age groups: 0–5 years; 6–20 years; 21–40 years; 41–60 years; 61–80 years; and ≥ 81 years. In effect, the monthly age-adjusted rate was the weighted average of the age-specific (crude) rates. This allowed us to reduce the potential confounding effect of age.

### Statistical approach and analysis

Our main analytical tool involved AutoRegressive Integrated Moving Average (ARIMA) models. The work of George Box and Gwilym Jenkins in the 1970s popularized ARIMA models, whose precursors included the ARMA models of Peter Whittle (1951). They have had increasing use over the past few decades, with some minor development of the analytical approaches and increasing emphasis on model usefulness and interpretability ([23], p. 20). In recent years ARIMA models have had considerably increased use within the health sciences. The basic equation for a seasonal ARIMA model may be expressed as follows.

Here, *Y*_*t*_ represents the time series data at time *t*; *c* is a constant term for the intercept; *ϕ*_i_ are the *AR* coefficients for non-seasonal lags *i* = *1* to *p*; *Y*_*t-1*_ is the value of the time series at lag *i*; *θ*_*j*_ are the *MA* coefficients for non-seasonal lags *j* = 1 to *q*; *e*_*t-j*_ is the error term at lag *j* (difference between predicted and observed values); *S* represents the seasonality period; Φ are the seasonal *AR* coefficients at seasonal lags *s* = *1* to *S; Y*_*t-s*_ is the value of the time series at seasonal lag *s*; Θ_s_ are the seasonal *MA* coefficients for seasonal lags *s* = 1 to *S*; *e*_*t-s*_ is the error term at seasonal lag *s*; and *e*_*t*_ is the error term at the current time *t*.

In this study, the total monthly data points generated qualified this as a medium-length time series. This length increases reliability for analysis of intervention impact and allows for a seasonal component. The risk of model misspecification, and of type I and type II errors is inversely proportional to time series length ([23], p. 264).

A total of 13 ARIMA models were developed: one for each of the four readmission conditions within the P4P intervention; across three hospital sizes for each of general and pneumonia readmissions; and one for each of the three readmission conditions not included in the P4P intervention (myocardial infarction, cataract surgery and appendectomy). The non-P4P readmissions were selected because they occur frequently and are somewhat similar to the P4P readmission types. Hospitals were categorized as small (< 50 beds), medium (50–100 beds) or large (> 100 beds). We used an iterative identify-estimate-diagnose process, evaluating several models before narrowing down on each final model ([23], p. 19). We elaborate on the analytical process steps in Table 1. We opted not to use forecasted-to-observed difference, to avoid the errors associated with this approach ([23], p. 167). We also undertook sensitivity analysis by constructing ARIMA models using a hypothetical month intervention for each of the three months before and after the actual intervention. For further comparison purposes, we estimated a single-group interrupted time series analysis with Newey ordinary least-squares (OLS) regression, including seasonality adjustment. Two-sided statistical significance was set at *p* < 0.05.

**Table 1:** Analytical process steps

Step	Description
1	A priori, we expected the intervention to (potentially) have an immediate impact on the scale of weeks, more likely resulting in a level change in readmission rates, but possibly slope and/or pulse changes. We did not identify other policies or interventions that may have otherwise affected our outcome measure
2	We generated descriptive statistics, and identified eight gaps in the data (expected) for each December throughout 2011–2019. This was due to MoPH IT data extraction limitations. We used interpolation to compute the readmission rates for these months, based on the average of the previous and subsequent month
3	We plotted monthly data points to visualize the data, and to support the identification of trends and outliers. Subsequent steps 4 to 6 used the pre-intervention data-points only
4	To determine stationarity of the series, we assessed if variance was changing over time (heteroscedasticity). Failure to transform a non-stationary series would be a threat to statistical conclusion validity, and renders unusable the subsequent steps and any conclusions ([23](p. 61)). We first used the Breusch-Pagan/Cook-Weisberg test. This was followed by the Dickey-Fuller test for unit root (non-stationarity). If a stationarity was found, then a differencing order would be applied to the data and iterative diagnostics repeated till a stationary series is produced
5	We tabulated and visualized autocorrelation and partial autocorrelation functions (ACF and PACF), to assess autocorrelation and stationarity, as well as inform the appropriate AR and/or MA terms of our final model. Since seasonality is expected, by the nature of the data, seasonal ARIMA models on the order of 12 months were pursued
6	We used a statistical command (autoarima) as a starting point to suggest models, following by individual model evaluation, using information developed from the previous steps. We pursued the most parsimonious model, dropping non-significant parameters while also relying on the Bayesian information criterion (BIC) generated. This was to avoid having unnecessary parameters resulting in increased variance and an over-fit model. The most practically useful model was chosen
7	We created the intervention variables for level, ramp and pule changes, as there was uncertainty regarding what impact type to expect. A priori, a level change was considered more likely. A pulse change was defined as a three-month change (1) which then reverted to baseline form (0). The model was estimated, with parsimony again pursued. A visualization of the data (from step 3) was also used to support interpretation
8	We ran diagnostics on the model, to ensure that the model met the assumptions upon which our analytical approach was based, specifically by comparing the residuals to white noise, and then testing for normality and independence. Residual plots were made for autocorrelations, partial autocorrelations, and a scatter plot to ensure non-heteroscedasticity. For normality testing, we used kernel density estimate plot, followed by quantile and standardized normal probability plots (Q-Q plot and P-P plot). If a normal distribution is approximated, then the residuals are considered to be no different than white noise. We then used the Kolmogorov–Smirnov to compare the outcome variable to a normal distribution. Testing for independence first involved a scatter-plot of residuals by time variable, followed by the Ljung-Box test on the residuals. Once normality and independence criteria were met, we considered the model to be statistically adequate, and the model selection algorithm is concluded. If any criteria were not met, the iterative process of model selection was repeated

## Results

The study population fulfilling the inclusion and exclusion criteria varied by readmission type (see Table 2). General readmissions included about 1.33 million hospitalizations across 2011–2019, and 80,080 readmissions. Among P4P readmissions, index hospitalizations were lowest among stroke cases. Overall readmission rates for P4P conditions ranged from 2.54% (cholecystectomy) to 6.55% (stroke). Variability in monthly readmissions was greatest for stroke (standard deviation [SD] 2.31%) and least for general readmissions (SD 0.51%). The monthly readmission rate reflects month-to-month variability, capturing temporal fluctuations, while the overall readmission rate represents the aggregate proportion of readmissions across the entire study period (or the pre- and post-intervention periods). The number of hospitals having different readmissions varied by year, but was generally similar across 2011–2019 (see Additional file 1, Supplement B). About twice as many hospitals had pneumonia and general readmissions than those that had cholecystectomy and stroke readmissions.

**Table 2:** Descriptive statistics of 30-day readmissions for four condtions, 2011–2019

		**General cases**	**Pneumonia**	**Cholecystectomy**	**Stroke**	**Myocardial infarction**	**Cataract surgery**	**Appendectomy**
**Total admissions**		1,333,691	70,585	26,820	13,370	4,517	51,073	10,803
**Total readmissions**		80,080	3,569	681	876	324	3,348	237
**Overall readmission rate**	**Mean**	6.00%	5.06%	2.54%	6.55%	7.17%	6.56%	2.19%
	**SD**	0.24%	0.22%	0.16%	0.25%	0.26%	0.25%	0.15%
**Pre-intervention**	**Admissions**	1,087,952	58,452	21,607	10,789	3,737	39,983	8,990
	**Readmissions**	64,977	2,886	577	714	254	2,496	210
	**Mean, *(95% CI)***	5.97% *(5.93%—6.02%)*	4.94% *(4.76%—5.12%)*	2.67% (2.45%—2.89%)	6.62% *(6.14%—7.10%)*	6.80% *(5.97%—7.62%)*	6.24% *(6.00%—6.48%)*	2.34% *(2.02%—2.65%)*
**Post-intervention**	**Admissions**	245,739	12,133	5,213	2,581	780	11,090	1,813
	**Readmissions**	15,103	683	104	162	70	852	27
	**Mean, *(95% CI)***	6.15% *(6.04%—6.25%)*	5.63% *(5.19%—6.07%)*	2.00% *(1.59%—2.40%)*	6.28% *(5.27%—7.28%)*	8.97% *(6.82%—11.12%)*	7.68% *(7.15%—8.21%)*	1.49% *(0.89%—2.09%)*
**Monthly readmission rate**	**Mean**	5.91%	4.81%	2.42%	6.48%	7.19%	6.48%	2.08%
	**SD**	0.51%	0.94%	1.03%	2.31%	4.23%	1.50%	1.43%
	**Median**	5.87%	4.74%	2.36%	6.34%	6.95%	6.38%	1.94%

A simple comparison of pre- and post-intervention mean readmissions among P4P conditions shows increased general and pneumonia readmissions, and decreased cholecystectomy readmissions. However, it is emphasized that these pre- and post-intervention means and their confidence intervals (CIs) do not determine the intervention's impact due to the different seasonal distribution of data and the number of observations. Rather, the intervention’s effect is determined by the ARIMA models, which account for these factors, including seasonality and trends.

**Fig. 1:**
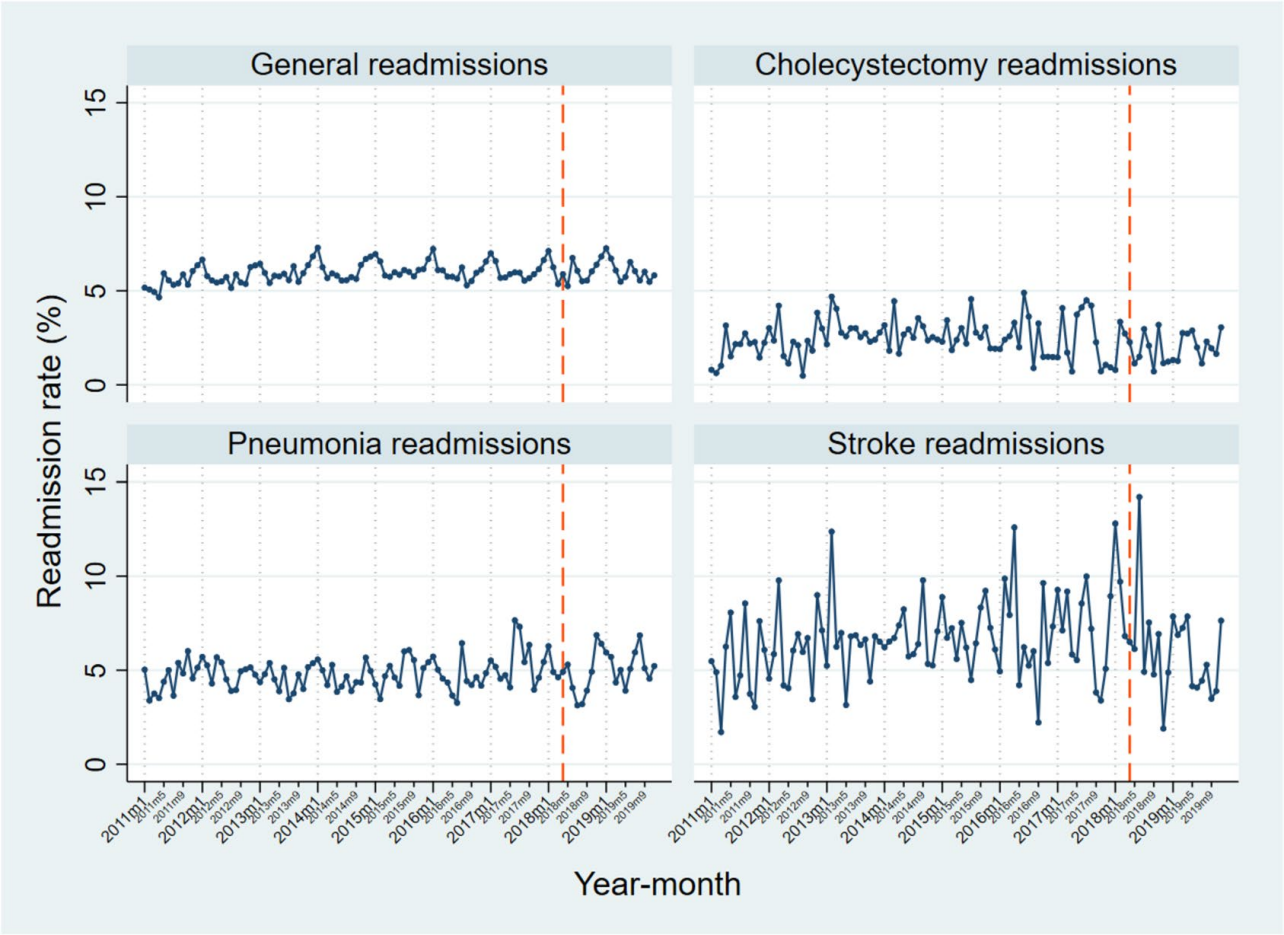
30-day readmission rates for P4P readmissions, 2011–2019, with dashed line representing the intervention time point

**Fig. 2:**
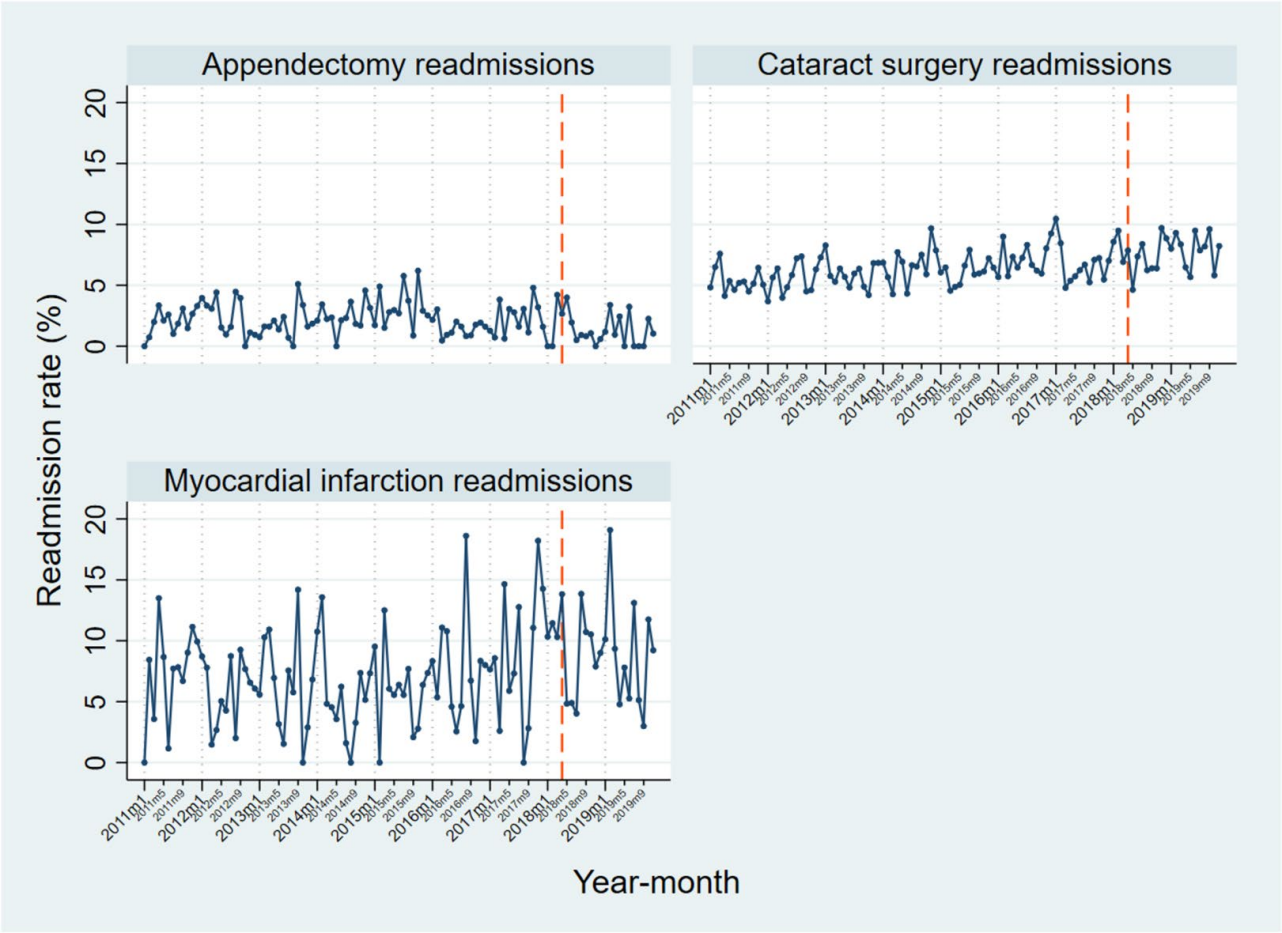
30-day readmission rates for non-P4P readmissions, 2011–2019, with dashed line representing the intervention time point

Seasonality was observable across readmissions, which further justified the a priori decision to use seasonal ARIMA models (see Figs. 1 and 2). Readmission rates data was stationary across all conditions. This was unsurprising for P4P readmissions, as one of the criteria used in their selection for P4P intervention had been that they displayed a generally stable long-term trend, to allow a better evaluation of change. The final ARIMA models are shown in Tables 3, 4, 5, and 6. A first-order seasonal difference was applied for all readmissions with one exception (general readmissions), as this provided the most practically useful model, in line with our analytical approach ([23] p83).

**Table 3:** Final ARIMA models and results across the four P4P readmission types, 2011–2019

	**General**			**Pneumonia**			**Cholecystectomy**			**Stroke**		
Model	(1,0,0) (1,0,0)_12_			(1,0,0) (1,1,0)_12_			(1,0,0) (1,1,0)_12_			(0,0,1) (0,1,1)_12_		
BIC	79.1			215.9			252.2			364.4		
Level coefficient, p, (95%CI)	0.256	0.075	(-0.026 to 0.537)	-0.154	0.658	(-0.837 to 0.528)	**-0.714**	0.048	(-1.420 to -0.008)	**-1.637**	0.012	(-2.907 to -0.367)
Constant	**5.825**	< 0.001	(5.571 to 6.080)	0.081	0.520	(-0.166 to 0.328)	0.084	0.450	(-0.134 to 0.302)	**0.274**	0.011	(0.063 to 0.485)
AR	**0.348**	< 0.001	(0.192 to 0.503)	**0.270**	0.037	(0.017 to 0.523)	0.071	0.585	(-0.184 to 0.326)	-	-	-
SAR	**0.664**	< 0.001	(0.510 to 0.817)	**-0.597**	< 0.001	(-0.774 to -0.420)	**-0.502**	< 0.001	(-0.708 to -0.296)	-	-	-
MA	-	-	-	-	-	-	-	-	-	-0.056	0.693	(-0.331 to 0.220)
SMA	-	-	-	-	-	-	-	-	-	**-0.870**	< 0.001	(-1.190 to -0.550)
Sigma	**0.344**	< 0.001	(0.299 to 0.388)	**0.973**	< 0.001	(0.866 to 1.080)	**1.188**	< 0.001	(1.018 to 1.358)	**2.281**	< 0.001	(1.924 to 2.638)
Log likelihood	-41.1	-	-	-134.9	-	-	-152.9	-	-	-221.1	-	-
Kolmogorov–Smirnov test	-	0.480	-	0.899		-	-	0.950	-	-	0.389	-
Ljung-Box test	-	0.806	-	0.739		-	-	0.949	-	-	0.900	-

*BIC* Bayesian information criterion, *(S)AR* (seasonal) autoregressive term, *(S)MA* (seasonal) moving average term

**Table 4:** Final ARIMA models and results across the three non-P4P readmission types, 2011–2019

	**Myocardial infarction**			**Cataract surgery**			**Appendectomy**		
Model	(1,0,0) (1,1,0)_12_			(0,0,1) (0,1,1)_12_			(0,0,1) (0,1,1)_12_		
BIC	79.1			351.3			385.9		
Level coefficient, p, (95%CI)	1.726	0.607	(-4.842 to 8.295)	0.167	0.891	(-2.223 to 2.557)	-1.255	0.523	(-5.105 to 2.594)
Constant	0.354	0.378	(-0.432 to 1.140)	**0.265**	0.008	(0.071 to 0.460)	-0.002	0.983	(-0.150 to 0.147)
AR	0.021	0.850	(-0.197 to 0.239)	-	-	-	-	-	-
SAR	**-0.539**	< 0.001	(-0.724 to -0.353)	-	-	-	-	-	-
MA	-	-	-	**0.289**	0.011	(0.066 to 0.511)	0.066	0.554	( -0.153 to 0.286)
SMA	-	-	-	**-0.650**	< 0.001	(-0.910 to -0.390)	-0.998	0.943	(-28.446 to 26.451)
Sigma	**4.818**	< 0.001	(4.202 to 5.434)	**1.255**	< 0.001	(1.056 to 1.453)	1.359	0.443	(0 to 19.896)
Log likelihood	-286.2	-	-	-159.7	-	-	-177.0	-	-
Kolmogorov–Smirnov test	-	0.947	-	0.601		-	-	0.597	-
Ljung-Box test	-	0.971	-	0.626		-	-	0.832	-

**Table 5:** Final ARIMA models for general readmissions among small, medium and large hospitals, 2011–2019

**General readmissions**	**Small hospitals**			**Medium hospitals**			**Large hospitals**		
Model	(1,0,0) (1,0,0)_12_			(1,0,0) (1,0,0)_12_			(1,0,0) (1,0,0)_12_		
BIC	114.2			115.7			235.7		
Level coefficient, p, (95%CI)	0.246	0.168	(-0.104 to 0.596)	-0.044	0.801	(-0.384 to 0.297)	0.289	0.331	(-0.294 to 0.872)
Constant	**5.951**	< 0.001	(5.689 to 6.211)	**5.727**	< 0.001	(5.510 to 5.944)	**5.442**	< 0.001	(5.156 to 5.728)
AR	**0.299**	0.001	(0.128 to 0.470)	**0.369**	< 0.001	(0.201 to 0.538)	**0.228**	0.020	(0.036 to 0.419)
SAR	**0.667**	< 0.001	(0.523 to 0.811)	0.209	0.089	(-0.032 to 0.449)	0.157	0.179	(-0.072 to 0.386)
MA	-	-	-	-	-	-	-	-	-
SMA	-	-	-	-	-	-	-	-	-
Sigma	**0.403**	< 0.001	(0.340 to 0.465)	**0.473**	< 0.001	(0.411 to 0.535)	**0.838**	< 0.001	(0.717 to 0.958)
Log likelihood	-58.1	-	-	-72.1	-	-	-133.0	-	-
Kolmogorov–Smirnov test	-	0.430	-	0.993		-	-	0.366	-
Ljung-Box test	-	0.965	-	0.540		-	-	0.918	-

*BIC* Bayesian information criterion, *(S)AR* (seasonal) autoregressive term, *(S)MA* (seasonal) moving average term

**Table 6:** Final ARIMA models for pneumonia readmissions among small, medium and large hospitals, 2011–2019

**Pneumonia**	**Small hospitals**			**Medium hospitals**			**Large hospitals**		
Model	(1,0,0) (1,0,0)_12_			(1,0,0) (1,1,0)_12_			(0,0,1) (0,1,1)_12_		
BIC	267.6			342.8			468.8		
Level coefficient, p, (95%CI)	0.145	0.670	(-0.521 to 0.811)	-0.411	0.547	(-1.751 to 0.928)	-0.326	0.872	(-4.299 to 3.647)
Constant	**4.767**	< 0.001	(4.452 to 5.082)	0.107	0.563	(-0.255 to 0.468)	0.163	0.592	(-0.434 to 0.760)
AR	0.205	0.055	(-0.004 to 0.415)	0.085	0.411	(-0.118 to 0.288)	-	-	-
SAR	0.114	0.224	(-0.070 to 0.298)	**-0.531**	< 0.001	( -0.722 to -0.338)	-	-	-
MA	-	-	-	-	-	-	**0.177**	0.031	(0.016 to 0.338)
SMA	-	-	-	-	-	-	**-0.790**	< 0.001	(-1.018 to -0.561)
Sigma	**1.036**	< 0.001	(0.873 to 1.199)	**2.098**	< 0.001	(1.798 to 2.399)	**1.188**	< 0.001	(1.018 to 1.358)
Log likelihood	-155.7	-	-	-207.2	-	-	-281.3	-	-
Kolmogorov–Smirnov test	-	0.910	-	0.755		-	-	0.029	-
Ljung-Box test	-	0.872	-	0.851		-	-	0.750	-

*BIC* Bayesian information criterion, *(S)AR* (seasonal) autoregressive term, *(S)MA* (seasonal) moving average term

The first iterations of the intervention models included all three changes (level, ramp, pulse), which we subsequently narrowed down by parsimony and data visualization. The final intervention models included a level change alone. Independence and normality criteria were met by all final models, as assessed in the post-estimation diagnostic tests and plots (see Tables 3, 4, 5 and 6 and Additional file 1, Supplement C).

Using the ARIMA intervention models, there was no evidence of intervention impact on two of the P4P readmission conditions (general and pneumonia) and on all three of the non-P4P conditions (myocardial infarction, cataract surgery and appendectomy). However, a level change was found in both cholecystectomy and stroke readmissions following the intervention. Mean monthly cholecystectomy readmissions decreased by 5.9% (0.1%-11.8%) and stroke readmissions decreased by 13.6% (3.1%-24.2%), which were calculated by dividing the coefficients by 12 to account for seasonality. Tables 3, 4, 5 and 6 summarize the results of all final ARIMA models.

Validation using Newey OLS regression found a decreased level change for cholecystectomy readmissions by about 52.7% (6.3%-99.0%, *p* = 0.026), but no change was found among the remaining six conditions. Sensitivity analysis was conducted using ARIMA models to assess for change from a hypothetical intervention, for every month between December 2017 and July 2018 for the P4P readmissions (see Table 7). A decrease in monthly readmissions was found for cholecystectomy when using the hypothetical intervention months of April and May 2018, and for stroke when using the months between February and July 2018, but no change was found for readmissions of pneumonia and general cases (except of June 2018). Repeating sensitivity analysis using Newey OLS regression, we did not find change among general and pneumonia readmissions.

**Table 7:** Sensitivity analysis of ARIMA results^a^ across four readmission types using hypothetical intervention points, 2011–2019

**Hypothetical intervention**	**General cases**			**Pneumonia**			**Cholecystectomy**			**Stroke**		
**Model**	(1,0,0) (1,0,0)_12_			(1,0,0) (1,1,0)_12_			(1,0,0) (1,1,0)_12_			(0,0,1) (0,1,1)_12_		
	**Coeff**	***p***	**95% CI**	**Coeff**	***p***	**95% CI**	**Coeff**	***p***	**95% CI**	**Coeff**	***p***	**95% CI**
**December 2017**	0.105	0.446	(-0.165 to 0.374)	-0.136	0.699	(-0.826 to 0.554)	-0.708	0.064	(-1.458 to 0.042)	-0.665	0.333	(-2.011 to 0.681)
**January 2018**	0.104	0.439	(-0.159 to 0.366)	-0.110	0.755	(-0.797 to 0.578)	-0.641	0.100	(-1.404 to 0.122)	-0.905	0.188	(-2.252 to 0.442)
**February 2018**	0.046	0.730	(-0.217 to 0.310)	-0.233	0.504	(-0.917 to 0.450)	-0.538	0.174	(-1.312 to 0.237)	**-1.480**	0.026	(-2.780 to -0.180)
**March 2018**	0.107	0.396	(-0.140 to 0.353)	-0.125	0.723	(-0.814 to 0.565)	-0.685	0.060	(-1.398 to 0.028)	**-1.676**	0.011	(-2.963 to -0.390)
April 2018*	0.256	0.075	(-0.026 to 0.537)	-0.154	0.658	(-0.837 to 0.528)	**-0.714**	0.048	(-1.420 to -0.008)	**-1.637**	0.012	(-2.907 to -0.367)
**May 2018**	0.084	0.443	(-0.130 to 0.298)	-0.125	0.709	(-0.781 to 0.531)	**-0.695**	0.045	(-1.376 to -0.014)	**-1.563**	0.015	(-2.821 to -0.305)
**June 2018**	**0.323**	0.009	(0.082 to 0.564)	-0.435	0.205	(-1.109 to 0.239)	-0.516	0.165	(-1.244 to 0.212)	**-1.672**	0.010	(-2.948 to -0.395)
**July 2018**	0.123	0.471	(-0.212 to 0.458)	-0.428	0.160	(-1.027 to 0.170)	-0.243	0.550	(-1.040 to 0.554)	**-2.393**	0.002	(-3.935 to -0.850)

^a^Actual intervention, which was used in the primary analysis

## Discussion

Our study suggests that the addition of readmissions to hospital pay-for-performance resulted in a decrease of stroke and cholecystectomy readmissions. We found no evidence of impact neither on pneumonia and general case readmissions (across all hospital sizes), nor on myocardial infarction, cataract surgery and appendectomy readmissions (non-P4P).

Several factors may have contributed to our findings. These include intervention design, context and characteristics of patients and hospitals. Intervention design has a major role in determining outcomes. A narrative review of the US Medicare programs structured this under four domains: program scope (broad, narrow), performance (relative, absolute), awards (achievement, improvement, both), and incentive (reward, penalty, both) [26]. The Lebanese MoPH’s P4P model had a mixed scope, using relative hospital performance, and rewarding achievement. Hospitals were incentivized to improve performance to achieve higher reimbursement tiers, with a higher tier translating into a 10–15% greater reimbursement, depending on case type [1]. This reimbursement-performance linkage has previously led to improved hospital performance in 2014, specifically by decreasing unnecessary hospitalization and improving coding practices [1]. Readmissions were a new component added to the P4P model in 2018. Each of the four readmissions types would equally contribute to the 2% of the total performance score. The readmission component may have been too small to incentivize hospitals to decrease certain readmissions, since the major TPS components were on patient satisfaction (20%), hospital accreditation (30%) and casemix (45%).

The number of hospitals that had cholecystectomy and stroke readmissions was about half of those that had pneumonia and general readmissions. Improvement across a few hospitals may be more easily reflected in national-level outcomes of the first group. Hospitalization volume per condition may also have a similar effect, as general cases were orders of magnitude greater than other conditions. In our study, the decrease in cholecystectomy and stroke readmission was not reflected at the scale of general readmissions. This may be due to a dilution effect, or mixed changes in non-targeted readmissions.

Readmission risk is also influenced by the medical complexity. Persons undergoing a relatively less complex surgery such as cholecystectomy are generally expected to be younger and healthier than those hospitalized with pneumonia or stroke. Patient characteristics such as comorbidities are not modifiable by hospitals. Therefore, the potential improvement space would vary across readmission types. Here we note the wider variation in readmission rates of stroke and cholecystectomy.

Broad outcome-based payments generally have more positive effects on quality and cost, compared to narrow models [27]. Using a similar logic, the P4P’s inclusion of general readmissions was intended to incentivize hospitals to address processes that could impact readmissions across a wide range of conditions. However, our study found no evidence of such change. This suggests that hospitals were unable and/or uninterested to more broadly address readmissions. Being broader than the three other readmission types, general readmissions are likely more challenging to address. Hospitals may have also considered the more specific readmissions as ‘low-hanging fruits’.

We also found no evidence of change in pneumonia and general readmissions, across small, medium and large hospitals. Such segregated analysis was not undertaken for the lower-volume conditions, due to decreased reliability. Previous studies in the US have found a mixed or weak associations between hospital size and readmission rate [10, 28, 29]. Some have suggested that increased bed capacity induces demand and makes readmissions easier, thus leading to more care, but not necessarily better care [28]. Hospital volume is related to hospital size, and has generally stronger associations with readmission rates. Higher volume is typically related to better outcomes, although some studies have found the opposite relation [30]. Among surgical conditions, higher volume has been associated with lower readmission rates, possible due to larger hospitals having more systematic approaches to determine when to discharge patients and prevent readmissions [10]. In the Lebanese context, we expected hospital size to be more relevant than volume, because the MoPH also contracts with the largest hospitals, but for a small volume of typically more complex patients.

There was no evidence of change in myocardial infarction, cataract surgery and appendectomy readmissions, all of which were not part of the P4P intervention. This suggests that hospitals that responded to the P4P intervention specifically focused on reducing readmissions for cholecystectomy and stroke, and/or did not make effective changes to reduce other types of readmissions.

### Strengths and limitations

Country-level experiences linking readmissions to financial incentives are few, and studies are predominantly reliant on the US experience. Our study contributes to this limited evidence base, and from a limited-resource country setting. We have developed readmission algorithms for four conditions, using routine administrative data spanning nine years and involving about 140 hospitals. Our analytical approach involved an established iterative stepwise process. Our use of stable medium-length time-series increases the validity of statistical conclusions reached. We were able to risk-adjust for age, which is the predominant risk factor for readmissions, but not for comorbidities, due to the lack of availability of such data from the MoPH. The role of the latter was minimized by including only one readmission per patient per year.

We cannot determine the extent to which lower readmissions genuinely reflect better quality of care. While we consider it probable that improved care resulted in decreased readmissions, other contributing factors are plausible. Some hospitals may have intentionally reduced access to hospitalization, either by treating in emergency rooms (ER) or otherwise refusing to re-hospitalize. The MoPH does not offer ER coverage for its beneficiaries and lacks the data to verify such practice. Although we are unable to estimate this factor’s contribution, we do not expect this to have had a major role, as decreases in readmissions were limited to two of the four conditions. There have been mixed findings of unintended consequences of the US HRRP experience. Earlier reports failed to find an association of hospital visits with readmission rates, while subsequent ones found decreased readmissions were accompanied by increased emergency and observation room stays [16, 31]. For example, up to 80% of the decrease in heart failure readmissions may be accounted for by increased emergency and observation room stays [19]. One solution would be to include the entire spectrum of hospital visits within a single outcome measure [19].

As is typical of hospital P4P, for the Lebanese MoPH initiative the hospital functions as a ‘black box’, into which we have little insight regarding the motivations, decisions and actions of managers and health professionals. Understanding the factors involved in mediating impact may allow us to better design P4P interventions, and also explain the differential impact observed across the readmission types included [32].

We are unable to assess any changes in post-discharge mortality, which may be another consequence of hospitals avoiding readmissions. Some studies of the US HRRP found an increase in post-discharge mortality among heart failure and pneumonia patients, but not those with acute myocardial infarction [18]. Other studies found increased mortality only among those hospitalized for heart failure [14]. In the US, an overly strong incentive for reducing readmissions has been noted, with the penalty for hospitals having excess readmissions being 15 times greater than those having excess mortality [14]. Increasing mortality for heart failure remains not fully understood, but this has led to recommendations that readmission and mortality be combined [19]. In Lebanon, the incentive for reducing readmissions is considerably smaller in scale than that of the US HRRP.

Competing risk models have been recently proposed as a solution to some unintended consequences. This is defined as an event whose occurrence impedes the occurrence of the primary outcome of interest [33]. For example, a readmissions competing risk model would keep any deaths that occur in the calculation denominator, rather than remove them as is standard in current metrics. For high-risk groups or conditions, such models would have greater relevance, given the more frequent mortality.

### Policy implications

Based on our findings and reflections using the wider literature, several recommendations can be offered for the P4P initiative in Lebanon. ER visits should be captured in the MoPH hospitalization data, and readmissions and mortality data should be linked. Readmission outcomes should be widened to include ER visits, and either combined to capture mortality, or within a competing risk model. The MoPH may consider alternating different readmission types within P4P, while excluding high-risk conditions or those more prevalent in more vulnerable groups (e.g. elderly). The focus on readmissions may be exchanged with processes directly related to them, such as hospital-to-community patient transitions.

## Conclusion

Engaging hospitals on pay-for-performance was followed by a decrease in stroke and cholecystectomy readmissions. We did not find a change in pneumonia and general readmissions, regardless of hospital size. We also did not find a change in myocardial infarction, cataract surgery and appendectomy readmissions, all of which were not part of the P4P intervention. Including readmissions within P4P has the potential to improve patient outcomes and system efficiency. Encompassing all hospital visit types and capturing patient mortality would help mitigate unintended consequences. It is conceivable that the next generation of P4P may use a competing risk design, and exchange complex outcomes with processes known to favorably influence them.

## Supplementary Information

Supplementary Material 1.

Supplementary Material 2.

## Data Availability

The source data are owned by the Lebanese Ministry of Public Health and in line with the IRB approval granted for this study, the authors are not permitted to share the source patient-level data. In compliance with the MoPH’s obligation on data privacy, the underlying data are accessible in a de-identified form upon request to the Ministry of Public Health (directorgeneral@moph.gov.lb), including a justification for the request. The readmission rates generated using the developed algorithms and analyzed in this study are included in Additional file 2 of this article.

## Abbreviations

ACF: AutoCorrelation Function
AR: AutoRegressive term
ARIMA: AutoRegressive Integrated Moving Average
BIC: Bayesian Information Criterion
BPT: Hip Fracture Best Practice Tariff (England)
COPD: Chronic Obstructive Pulmonary Disease
CPT: Current Procedural Terminology
ER: Emergency Rooms
HRRP: Hospital Readmission Reduction Program (United States)
ICD10: Tenth revision of the International Statistical Classification of Diseases and Related Health Problems
IRB: Institutional Review Board
ITS: Interrupted Time Series
MA: Moving Average term
MoPH: Ministry of Public Health
OLS: Ordinary Least Squares
P4P: Pay-for-Performance
PACF: Partial AutoCorrelation Function
SAR: Seasonal AutoRegressive term
SMA: Seasonal Moving Average term
