# Supplementary material for: Impact of pay-for-performance on hospital readmissions in Lebanon: an ARIMA-based intervention analysis using routine data
Source: BMC Health Serv Res. 2024 Dec 5;24:1551. doi: 10.1186/s12913-024-12045-1 (PMC11619647; doi:10.1186/s12913-024-12045-1)

# Supplement A

**Data development details**

The case definitions for each of the readmission measures specified the inclusion criteria (diagnosis and procedure codes), and exclusion criteria (cardiac catheterization, lithotripsy, renal dialysis, chemotherapy, radiotherapy, malignancy, obstetric cases, motor vehicle accidents, blood transfusion, palliative care and transfer/same-day cases).

Since the data was provided with an annual timeframe by the MoPH, admissions in December had been artificially limited to an end-of-year discharge date. The calculation of monthly readmissions was therefore made for 11 months per year (January to November), including December cases only for identifying readmissions, and not for index hospitalization (i.e. for the numerator, not the denominator). This allowed the time opportunity for November cases to be readmitted in December.

**ARIMA models description**

ARIMA models include three components. An AutoRegressive (AR) component that indicates that an outcome variable is dependent on the previous values of this same term, and a stochastic term (randomness). A Moving Average (MA) component represents the influence of past error terms on the current error term; this is not to be confused with moving average of an outcome variable). In essence, an AR process remembers past realizations, while an MA process remembers past shocks. The Integrated (I) component indicates a transformation (integration) function which may be used to normalize the time series. The combination of these three components forms an ARIMA model.

All models require that the data be stationary. Where normality is violated (i.e. non-stationary), a normalization transformation of the data is necessary, to meet the underlying model assumptions. This pre-requisite is essential, because the analytical mechanism requires that a time series process operate identically in the future as it has in the past.

# Supplement B

**Table 8: Hospitals with readmissions, by hospital size, 2011-2019.**

| **Condition** | **Size** | **2011** | **2012** | **2013** | **2014** | **2015** | **2016** | **2017** | **2018** | **2019** |
| --- | --- | --- | --- | --- | --- | --- | --- | --- | --- | --- |
| **General cases** | <50 beds | 40 | 40 | 44 | 48 | 48 | 51 | 51 | 55 | 53 |
|  | 50-100 beds | 54 | 55 | 53 | 54 | 55 | 54 | 56 | 56 | 55 |
|  | 101-200 beds | 28 | 28 | 28 | 28 | 28 | 28 | 28 | 28 | 28 |
|  | >200 beds | 8 | 8 | 8 | 8 | 8 | 8 | 8 | 8 | 8 |
|  | **Total** | **130** | **131** | **133** | **138** | **139** | **141** | **143** | **147** | **144** |
|  |  |  |  |  |  |  |  |  |  |  |
| **Pneumonia** | <50 beds | 19 | 26 | 24 | 27 | 28 | 30 | 28 | 27 | 30 |
|  | 50-100 beds | 31 | 33 | 36 | 37 | 36 | 37 | 33 | 38 | 40 |
|  | 101-200 beds | 20 | 19 | 21 | 22 | 19 | 20 | 21 | 22 | 21 |
|  | >200 beds | 5 | 5 | 5 | 6 | 6 | 6 | 4 | 6 | 5 |
|  | **Total** | **75** | **83** | **86** | **92** | **89** | **93** | **86** | **93** | **96** |
|  |  |  |  |  |  |  |  |  |  |  |
| **Cholecystectomy** | <50 beds | 11 | 8 | 14 | 13 | 12 | 16 | 10 | 8 | 13 |
|  | 50-100 beds | 17 | 23 | 25 | 18 | 21 | 15 | 24 | 14 | 18 |
|  | 101-200 beds | 9 | 12 | 13 | 15 | 12 | 12 | 13 | 11 | 7 |
|  | >200 beds | 4 | 2 | 1 | 4 | 3 | 2 | 2 | 2 | 2 |
|  | **Total** | **41** | **45** | **53** | **50** | **48** | **45** | **49** | **35** | **40** |
|  |  |  |  |  |  |  |  |  |  |  |
| **Stroke** | <50 beds | 5 | 6 | 11 | 10 | 9 | 7 | 8 | 19 | 8 |
|  | 50-100 beds | 22 | 22 | 25 | 20 | 20 | 25 | 25 | 22 | 22 |
|  | 101-200 beds | 13 | 16 | 13 | 12 | 12 | 11 | 9 | 11 | 12 |
|  | >200 beds | 4 | 1 | 3 | 3 | 4 | 3 | 1 | 2 | 4 |
|  | **Total** | **44** | **45** | **52** | **45** | **45** | **46** | **43** | **54** | **46** |

# Supplement C

Diagnostic plots used in the analytical process are included below, using general readmissions data. This includes autocorrelation and partial autocorrelation plots of dependent variable and of residuals; kernel density plot; P-P plot and Q-Q plot; scatterplot of residuals and time period; and scatterplot of residuals and model dependent variable.


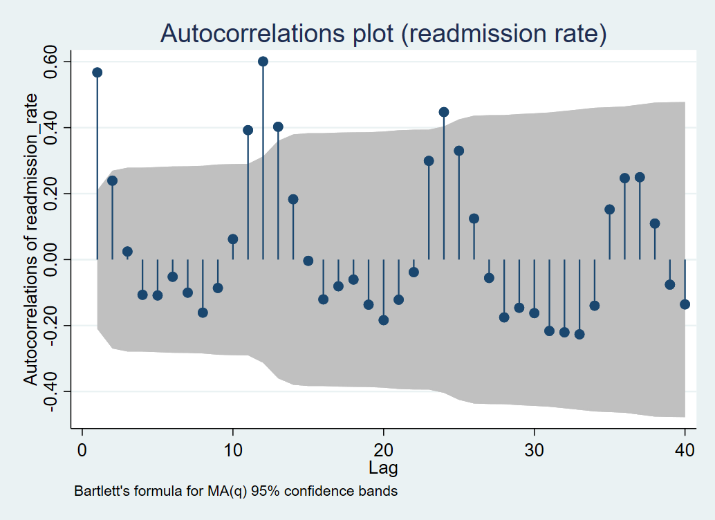

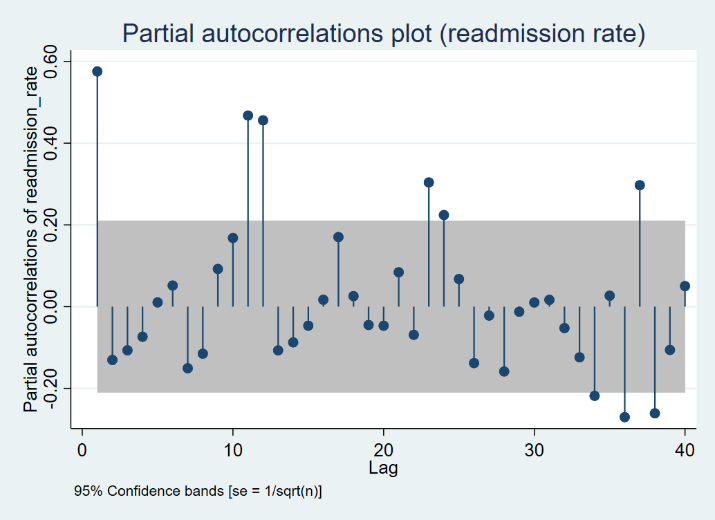

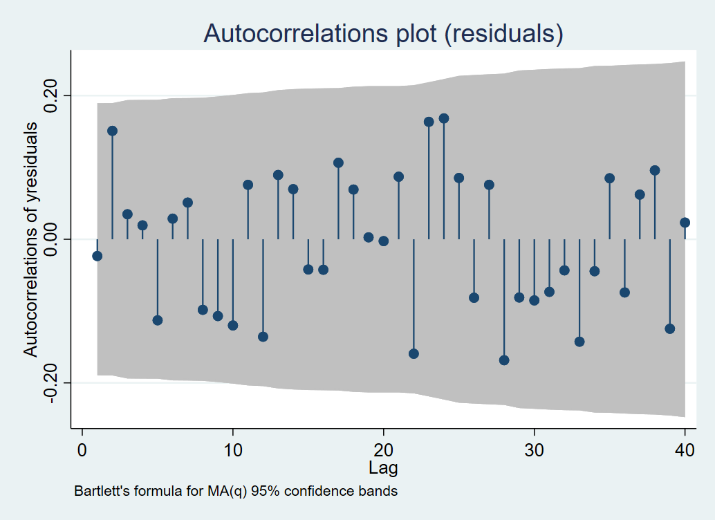

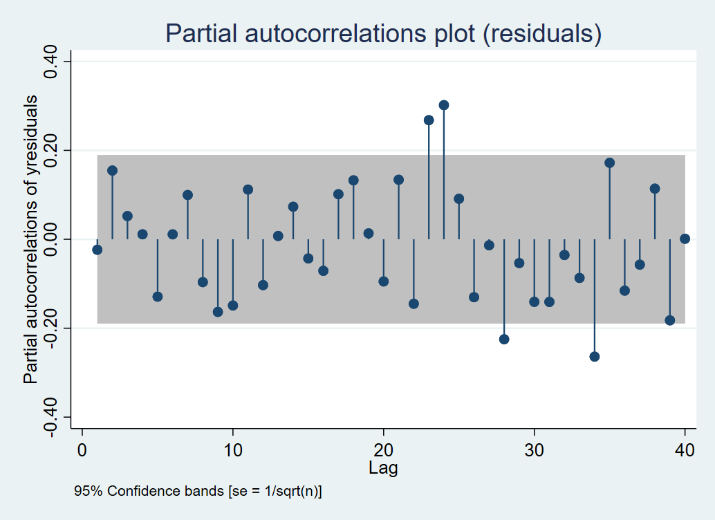

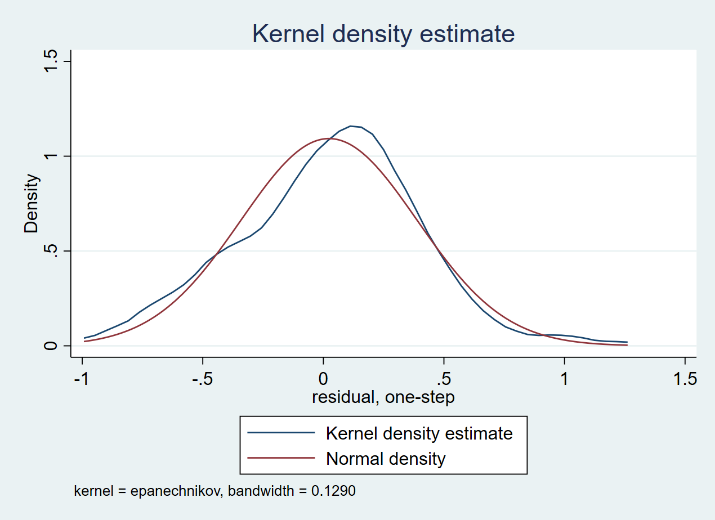

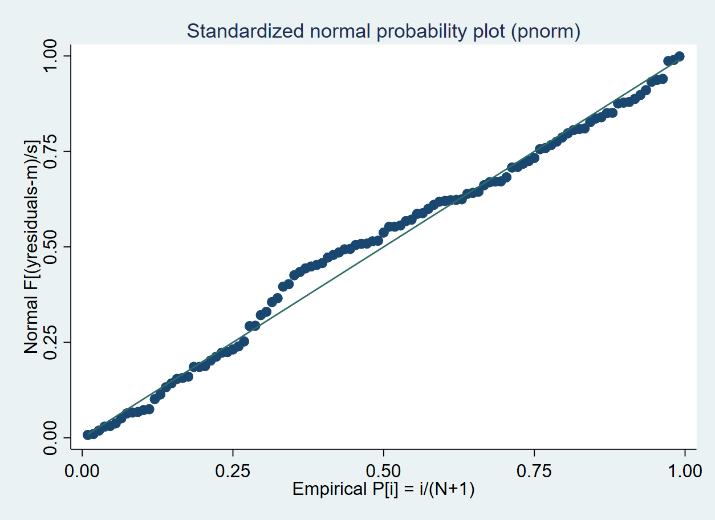

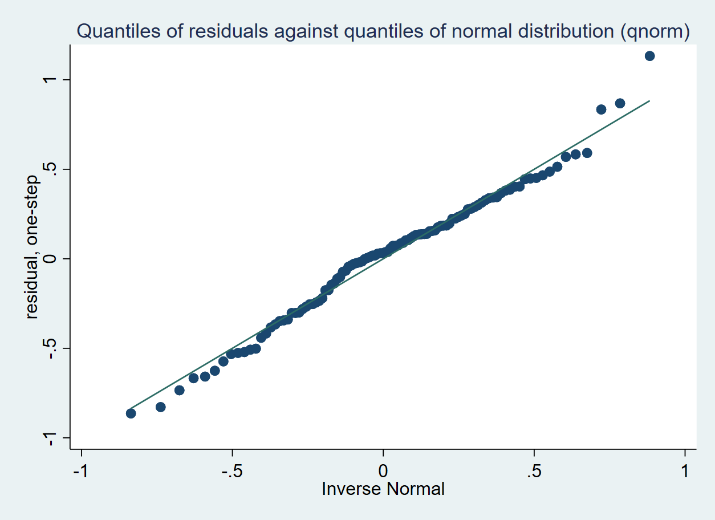

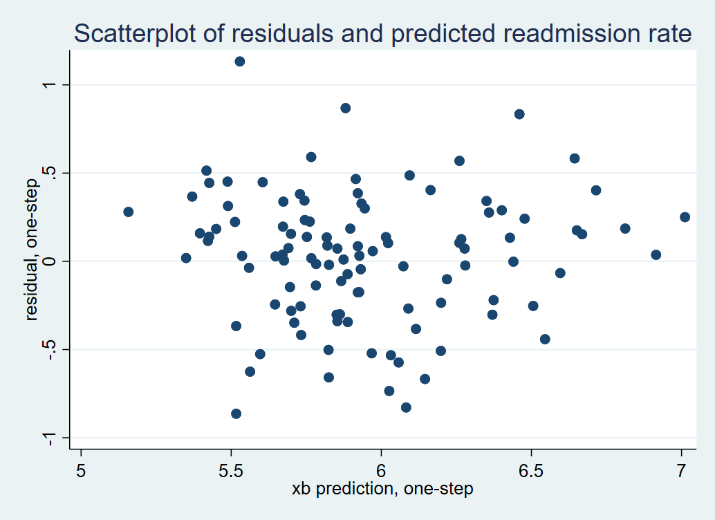

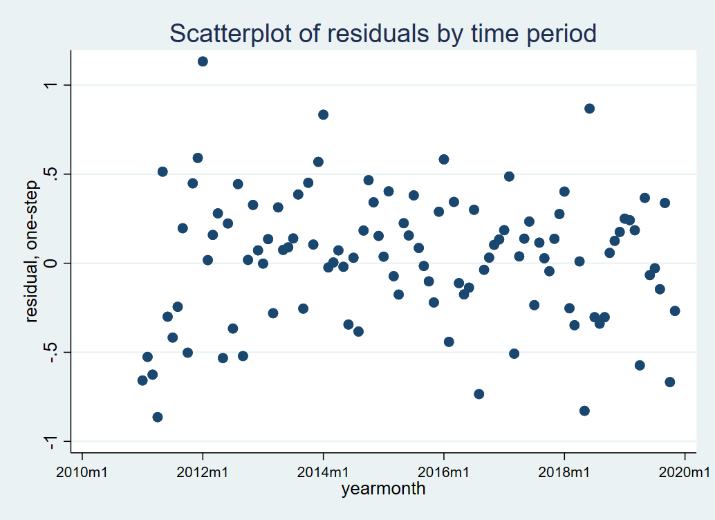

Supplement: Supplementary file 1 — Supplementary Material 1. [file 12913_2024_12045_MOESM1_ESM.docx]
